# Comparative Genome Microsynteny Illuminates the Fast Evolution of Nuclear Mitochondrial Segments (NUMTs) in Mammals

**DOI:** 10.1093/molbev/msad278

**Published:** 2023-12-20

**Authors:** Marek Uvizl, Sebastien J Puechmaille, Sarahjane Power, Martin Pippel, Samuel Carthy, Wilfried Haerty, Eugene W Myers, Emma C Teeling, Zixia Huang

**Affiliations:** Department of Zoology, National Museum, 19300 Prague, Czech Republic; Department of Zoology, Faculty of Science, Charles University, 12844 Prague, Czech Republic; Institut des Sciences de l’Evolution de Montpellier (ISEM), University of Montpellier, 34095 Montpellier, France; Institut Universitaire de France, Paris, France; School of Biology and Environmental Science, University College Dublin, Belfield, Dublin 4, Ireland; Max Planck Institute of Molecular Cell Biology and Genetics, 01307 Dresden, Germany; National Bioinformatics Infrastructure Sweden, Uppsala, Sweden; School of Biology and Environmental Science, University College Dublin, Belfield, Dublin 4, Ireland; Earlham Institute, Norwich Research Park, Colney Ln, NR4 7UZ Norwich, UK; School of Biological Sciences, University of East Anglia, Norwich, UK; Max Planck Institute of Molecular Cell Biology and Genetics, 01307 Dresden, Germany; School of Biology and Environmental Science, University College Dublin, Belfield, Dublin 4, Ireland; School of Biology and Environmental Science, University College Dublin, Belfield, Dublin 4, Ireland

**Keywords:** nuclear mitochondrial DNA segment (NUMT), genome microsynteny, evolution, mammal

## Abstract

The escape of DNA from mitochondria into the nuclear genome (nuclear mitochondrial DNA, NUMT) is an ongoing process. Although pervasively observed in eukaryotic genomes, their evolutionary trajectories in a mammal-wide context are poorly understood. The main challenge lies in the orthology assignment of NUMTs across species due to their fast evolution and chromosomal rearrangements over the past 200 million years. To address this issue, we systematically investigated the characteristics of NUMT insertions in 45 mammalian genomes and established a novel, synteny-based method to accurately predict orthologous NUMTs and ascertain their evolution across mammals. With a series of comparative analyses across taxa, we revealed that NUMTs may originate from nonrandom regions in mtDNA, are likely found in transposon-rich and intergenic regions, and unlikely code for functional proteins. Using our synteny-based approach, we leveraged 630 pairwise comparisons of genome-wide microsynteny and predicted the NUMT orthology relationships across 36 mammals. With the phylogenetic patterns of NUMT presence-and-absence across taxa, we constructed the ancestral state of NUMTs given the mammal tree using a coalescent method. We found support on the ancestral node of Fereuungulata within Laurasiatheria, whose subordinal relationships are still controversial. This study broadens our knowledge on NUMT insertion and evolution in mammalian genomes and highlights the merit of NUMTs as alternative genetic markers in phylogenetic inference.

## Introduction

Nearly all eukaryotic nuclear genomes have constantly been challenged by the insertion of foreign DNA of various origins, essentially shaping their architecture over evolutionary time (Adams et al. 2000; Richly and Leister 2004; Kleine et al. 2009). In mammalian genomes, the acquisition of extrinsic DNA is largely conducted by the horizontal transfer of mitochondrial DNA (mtDNA) segments, leading to nuclear pseudogenes of mitochondria origin (NUMTs; Mourier et al. 2001; Timmis et al. 2004; Hazkani-Covo et al. 2010). Although the mechanism of this ongoing process is not yet fully understood, the well-accepted hypothesis proposes that the passive uptake of mtDNA fragments into the nuclear genome occurs at double-stranded DNA breaks via nonhomologous end-joining repair machinery (Blanchard and Schmidt 1996). A few lines of evidence have suggested the association between nuclear mitochondrial DNA segment (NUMT) integrations and human diseases (Turner et al. 2003; Wei et al. 2022); however, they are commonly regarded as “dead on arrival” pseudogenes, as evidenced by the presence of stop codons, indels, and frameshifts caused by random mutation, and the differences in the genetic code between the nuclear genome and mitogenome (Perna and Kocher 1996). While arguably not functional, NUMTs have been responsible for many occasions of misinterpretations in mtDNA heteroplasmy detection (Albayrak et al. 2016), mitochondrial disease studies (Wallace et al. 1997; Yao et al. 2008), and phylogenetic placements (Sorenson and Quinn 1998; Thalmann et al. 2004; Li et al. 2016), due to their homology to mtDNA. Attempts should, therefore, be made to identify NUMTs in genomes in order to avoid erroneous conclusions in mtDNA-related research.

NUMTs have been investigated across a broad spectrum of vertebrates, spanning fish, amphibians, reptiles, birds, and mammals (Antunes and Ramos 2005; Calabrese et al. 2017; Liang et al. 2018; Hazkani-Covo 2022; Triant and Pearson 2022). Within the realm of mammals, numerous studies have revealed large variations in NUMT dynamics, including their radiation, genomic distribution, mtDNA origin, functionality, as well as their insertion time and rates (Hazkani-Covo et al. 2010). The genetic bases underlying these variations and NUMT evolutionary trajectories in a mammal-wide context are poorly understood. It has been suggested that NUMTs, the molecular fossils of ancestral mtDNA, can be potential genetic markers to infer phylogenetic relationships (Bensasson et al. 2001). However, the application is only limited to a few studies that focused on groups of species in narrow phylogenetic brackets, such as Primates (Hazkani-Covo and Graur 2007; Hazkani-Covo 2009), Passeriformes (Liang et al. 2018), and Chiroptera (Puechmaille et al. 2011). The major challenge resides in the assignment of NUMT orthology across mammals, owing to the rapid gain and loss of NUMTs (Hazkani-Covo et al. 2010), fast sequence changes (Hazkani-Covo et al. 2010), and considerable chromosome rearrangements over 200 million years of evolution (Pevzner and Tesler 2003). Currently, the primary method to predict orthologous NUMT loci across species is by means of aligning NUMT sequences along with their flanking regions or through whole genome alignments (Hazkani-Covo et al. 2010). These alignment-based approaches are only feasible to predict NUMT orthology within closely related species in which the noncoding genomic regions are well aligned (Wei et al. 2022). Hence, to elucidate NUMT evolution in mammals, it is imperative to develop new methods that enable NUMT orthology assignment between distantly related species.

Genomic synteny has been deeply conserved across the tree of vertebrates (Simakov et al. 2020). Microsynteny is defined as a fine-scale genomic region in which the order of a number of genes is evolutionarily conserved across species. It provides a valuable framework to interpret gene orthology relationships between species, especially for large multigene families or fast-evolving noncoding genes such as NUMTs (Pevzner and Tesler 2003). In this study, we comprehensively investigated the characteristics of NUMT insertions in 45 mammalian nuclear genomes and established a novel, synteny-based approach to accurately predict orthologous NUMTs and ascertain their evolution across mammalian clades. We observed that the Primate suborder Haplorhini has undergone a burst of NUMT insertions, while multiple NUMT expansion events may have occurred during the evolution of marsupials. Using comparative analyses across taxa, we showed that the mtDNA regions from which NUMTs originate are nonrandom. We also showed that NUMTs are likely found in transposon-rich and intergenic regions, and unlikely to code for functional proteins. Using this novel approach we established, we performed 630 pairwise comparisons of genome-wide microsynteny and assigned NUMT orthology relationships across 36 mammals. We further constructed the ancestral state of NUMTs using a coalescent approach and discovered that the phylogenetic patterns of NUMT presence-and-absence in Laurasiatheria support the ancestral clade of Fereuungulata. This challenges previous phylogenies which placed bats as the sister clade to ungulates, but agrees with the recent genome-based topologies which support a sister-group relationship between carnivores and ungulates. These results indicate that NUMT gain and loss over evolutionary time can provide new insights into mammal evolution. Given the conserved nature of genome synteny across vertebrates, our novel approach also holds the potential for extending its applications in the study of NUMT evolution across various taxa beyond mammals. However, we also demonstrated that one should be cautious when using ancestral NUMT trees to infer phylogenetic relationships. This study deepens our understanding of NUMT insertion and evolution in mammalian nuclear genomes and highlights the merit of NUMTs as alternative molecular markers in phylogenetic inference.

## Results

### Validity of NUMT Prediction

Using the optimal methods (see Materials and Methods), we obtained a landscape of NUMT insertions across 45 mammalian genomes (supplementary tables S1–S4, Supplementary Material online; supplementary fig. S1a, Supplementary Material online). It is a grand challenge to detect NUMTs, especially recent insertions, in mammalian genomes due to their homology to mtDNA and potential genome assembly errors that embed mtDNA sequences into nuclear genomes. With the exception of newly inserted NUMTs, authentic NUMTs usually exhibit a degree of sequence divergence from their mtDNA counterparts. We observed that, on average, only 2.96% of our predicted NUMTs have a higher than 98% sequence identity to their corresponding mtDNA across species (supplementary fig. S1b, Supplementary Material online). We also constructed 2 phylogenetic trees using the NUMT sequences that were mapped to mtDNA *CYTB* and *ND1* loci, alongside their native mtDNA sequences. In both trees, the NUMT sequences from each species exhibited unequivocal branching patterns separated from their corresponding mtDNA sequences, thereby providing further evidence of sequence divergence between NUMTs and their mtDNA counterparts (supplementary fig. S2, Supplementary Material online). Owing to data accessibility, we also explored our published PacBio raw reads for the genome assembly of the 6 bat species used in this study (see Materials and Methods). All the NUMTs predicted in these 6 species were located within individual PacBio reads where the flanking regions of the NUMT loci were mapped to the nuclear genome. The average coverage of PacBio reads spanning the junctions between NUMTs and nuclear DNA ranges from 13.3 to 15.8 (supplementary fig. S3a, Supplementary Material online). While it is difficult to distinguish newly inserted NUMTs from true mtDNA without Illumina or PacBio raw reads for genome assembly, these results indicate that our newly developed pipeline provides authentic and reliable NUMT predictions.

### Overview of NUMT Insertions in Mammalian Genomes

**Fig. 1.**
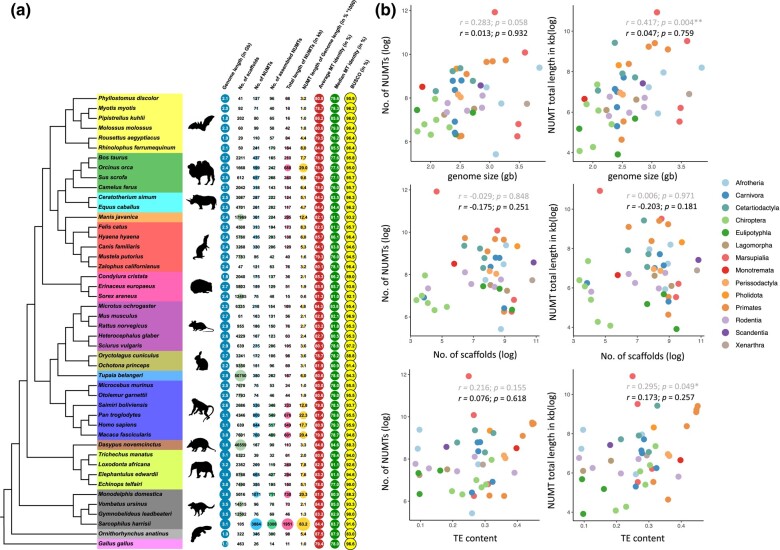
Overview of NUMTs in 45 mammalian genomes. a) Genome statistics and NUMT statistics in 45 mammalian species and the outgroup *Gallus gallus*. The phylogenetic relatedness of these 46 species was obtained from Jebb et al. (2020). The columns, from left to right, represent genome length (Gb), number of scaffolds, number of NUMTs, number of assembled NUMT blocks, cumulative NUMT length (kb), fraction (% × 1,000) of total NUMT length relative to genome length, average, and median NUMT sequence identity to the corresponding mtDNA, respectively, and BUSCO scores (%). The size of colored circles is scaled to the number displayed. b) Correlation between genome statistics and NUMT statistics. The scatterplots show the relationship between genome statistics (genome size, number of scaffolds, and average TE content) and NUMT statistics (number of NUMTs and accumulative NUMT length; log_2_ transformed). Correlation coefficients (*r*) and *P*-values were computed using Spearman's correlation test. In the scatterplots, coefficients (*r*) and *P*-values in light and dark shade indicate the values before and after phylogeny correction, respectively (*0.01 < *P* < 0.05, **0.001 < *P* < 0.01; see Materials and Methods).

The number of NUMT insertions ranges from 43 (manatee; *Trichechus manatus*) to 3,886 (Tasmanian devil; *Sarcophilus harrisii*), with the median of 218 (Fig. 1a). Our predictions are comparable to the numbers reported in previous studies (Hazkani-Covo et al. 2010; Calabrese et al. 2017; Hazkani-Covo 2022). Despite large variation in numbers across species, NUMTs only represent, on average, less than 0.01% of a genome (Fig. 1a). The genome of the Tasmanian devil (*S*. *harrisii*) carries the longest cumulative NUMT length (1,951 kb), while the shortest was found in the common shrew *Sorex araneus* (15.07 kb). The individual NUMT length varies between 30 bp and 16,699 bp across species, and exhibits a similar distribution in species within the same order (Kolmogorov–Smirnov test; supplementary fig. S3b and S3c, Supplementary Material online). Twenty of the forty-five genomes were found to possess exceptionally long NUMTs (over 10 kb), some of which were derived from almost the entire mtDNA (supplementary table S2, Supplementary Material online). Interestingly, the species in Cetartiodactyla have the highest percentages of complex NUMT blocks which comprise multiple individual NUMTs located within a genomic distance of 2 kb (see Materials and Methods; supplementary table S2, Supplementary Material online). To demonstrate the reliability of predicted NUMT blocks, as an example the genomic locus of the largest NUMT block in the *Molossus molossus* genome is supported by our published PacBio raw reads for the genome assembly (supplementary fig. S3d, Supplementary Material online). We noticed that the NUMT cumulative length illustrates a positive correlation with genome size (*P* = 0.004) and genome transposable element (TE) content (*P* = 0.049; specifically Long Interspersed Nuclear Elements [LINEs], *P* = 0.033), respectively (Spearman's correlation test; Fig. 1b; supplementary fig. S4, Supplementary Material online). However, cross-species correlational tests should take into account evolutionary relationships, and significances of the tests dropped after we corrected for the phylogeny of these 45 mammals (*P* = 0.759 and *P* = 0.257, respectively; Fig. 1b; supplementary fig. S4, Supplementary Material online). In addition to the similar NUMT length distribution observed among closely related species, these results suggest that NUMT insertions, to some degree, indicate mammal phylogeny and might be potential genetic markers for phylogenetic inference.

### NUMT Expansions in Primate and Marsupial Species

We observed a burst of NUMT insertions in Primates and Marsupialia. In Primates, 4 species with large NUMT numbers (547 to 846) lead to the node of the suborder Haplorhini, whereas the other 2 species studied in the sister suborder, Strepsirrhini, only have a small number of NUMT insertions (76 to 77; Fig. 1a). This suggests that a burst of NUMT insertions occurred in Haplorhini, after its divergence with Strepsirrhini. In Marsupialia, similar to our observation on the Tasmanian devil (*S. harrisii*), a recent study found that 2 species in the family Dasyuridae, the Tasmanian devil (*S. harrisii*) and the yellow-footed antechinus (*Antechinus flavipes*), had rapid NUMT expansions (Hazkani-Covo 2022). However, we also revealed that the opossum (*Monodelphis domestica*) in the family Didelphidae underwent a similar burst of NUMTs (1,083) as the Tasmanian devil, contrary to the possum *Gymnobelideus leadbeateri* (76) and the common wombat *Vombatus ursinus* (112) in the family Petauridae and Vombatidae, respectively (Fig. 1a). This result indicates that massive expansions of NUMT content may have occurred multiple times during the evolution of marsupials, and a thorough taxonomic sampling is crucial to drawing accurate conclusions on NUMT expansions.

### The mtDNA Regions From Which NUMTs Originate are Nonrandom

**Fig. 2.**
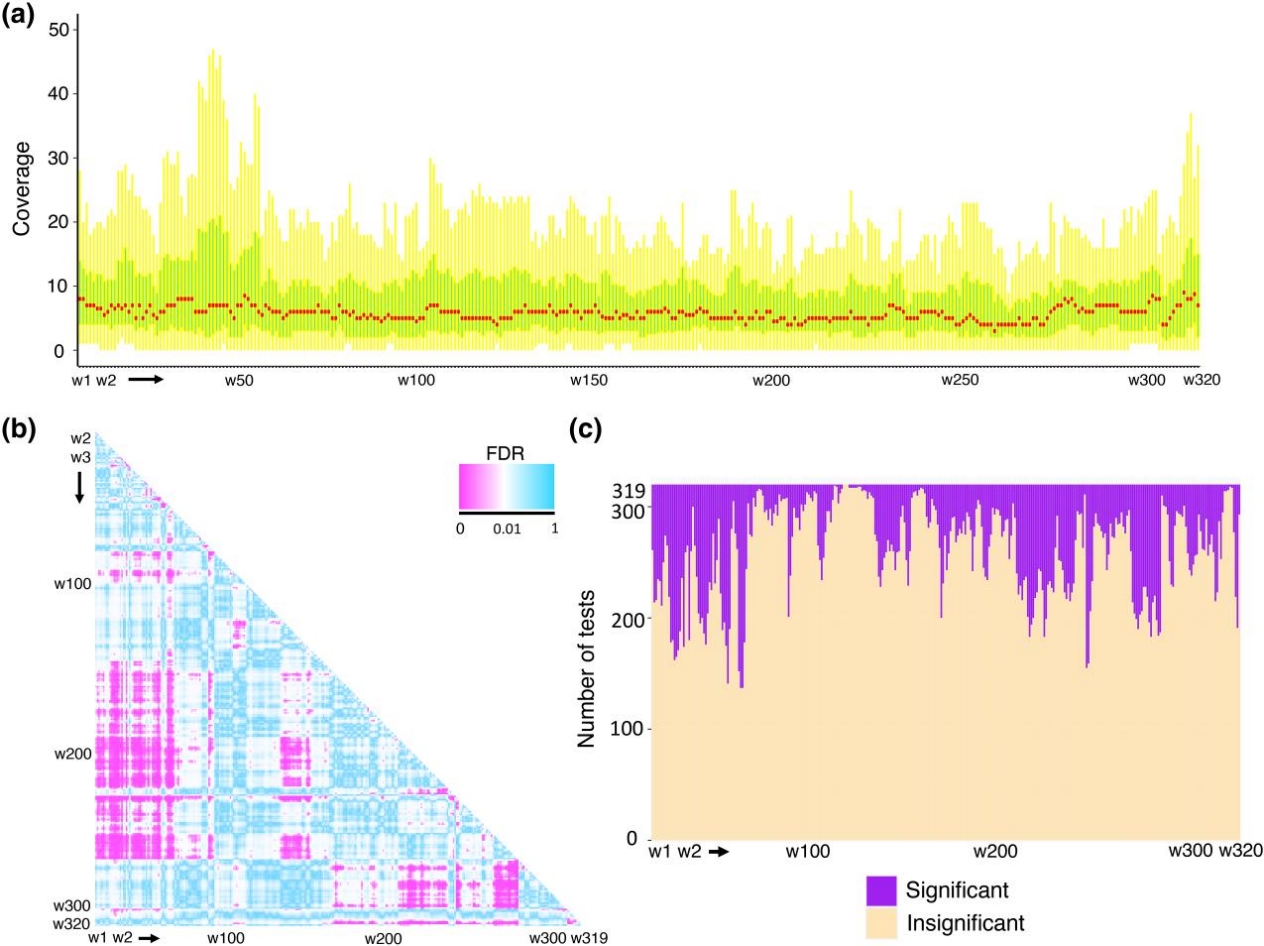
The mtDNA origin of NUMT sequences. a) Distribution of mtDNA coverage by NUMTs in a 50 bp window resolution. The *x*-axis represents 320 windows and the *y*-axis represents the coverage distribution of each window across 45 species. The median value of each window is highlighted in red, while the interquartile range is in green. The outliers are not shown in the boxplot. b) Pairwise comparisons of mtDNA coverage by NUMTs between 50 bp sliding windows using Mann–Whitney *U* test; 320 windows (w1 to w320) representing 16,000 bp in mtDNA were investigated. The heatmap depicts the FDR values of 51,040 tests between all possible windows. Pink colors indicate that the tests are significant (FDR < 0.01), while blue colors indicate that the tests are insignificant (FDR ≥ 0.01). c) Distributions of significant and insignificant testing results from pairwise comparisons between all possible windows. The coverage of each window across species was compared to those of the rest 319 windows, respectively.

It was reported that certain regions in mtDNA, such as the D-loop, tend not to produce NUMTs in a few primate genomes (Tsuji et al. 2012), while it is well represented by NUMTs in the cattle and a few cetacean genomes (Ko et al. 2015; Grau et al. 2020). By scanning the mitogenome with a 50 bp sliding window for each species (320 windows representing 16,000 bp in mtDNA per species), we conducted comparisons of mtDNA coverage by NUMTs between all possible windows across 45 species using pairwise Mann–Whitney *U* test (Fig. 2a–c; see Materials and Methods). We observed that the distribution of the NUMT coverage varies across windows (Fig. 2a). Of all 51,040 comparisons, 9,273 (18.17%) tests yielded significant results (false discovery rate, FDR < 0.01; Fig. 2b), and some windows (e.g. w1 to w50 corresponding to 1st to 2500th bp on mtDNA; w200 to 277 corresponding to 10,000th to 13,850th bp on mtDNA) illustrate different distributions of coverage from others (Fig. 2c). To further verify these results, we simulated coverage data for the null distribution of equal coverage by randomly reshuffling mtDNA coordinates of NUMTs for each species, performed pairwise comparisons across windows, and repeated these analyses 1,000 times (see Materials and Methods). We found that the number of significant tests yielded from the observed data (9,273) is ∼10 times as many as that of the simulated data (median: 953, supplementary fig. S5a, Supplementary Material online). This result supports our hypothesis that the mtDNA regions from which NUMTs originate are nonrandom. In addition, at single-nucleotide resolution, the mtDNA coverage of NUMTs exhibits a species-specific pattern across taxa (supplementary fig. S5b, Supplementary Material online). For each species, the mtDNA regions that are over-represented by NUMTs are listed in supplementary table S5, Supplementary Material online.

### NUMTs are Likely Found in Transposon-rich and Intergenic Regions

**Fig. 3.**
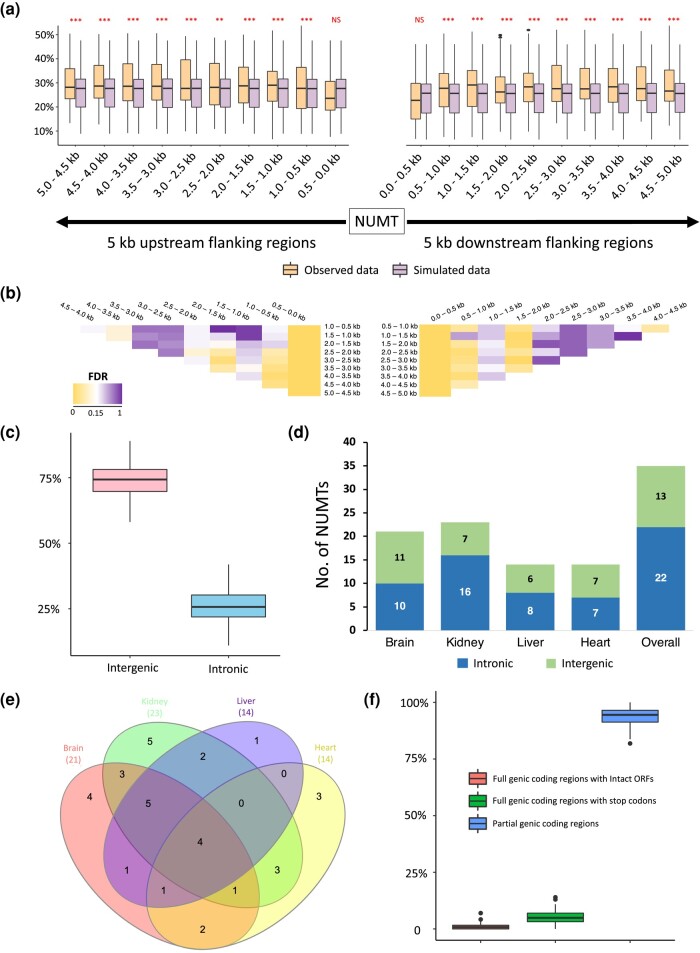
Characteristics of mammalian NUMTs. a) Transposable elements (TE) content in the 5 kb upstream and downstream of flanking regions of NUMTs/NUMT blocks with a window size of 500 bp. For each species, the average TE content was estimated for each of 20 windows in the flanking regions of all the NUMTs/NUMT blocks, and the same procedure was applied to the simulated data (background). For each window, the TE content between the observed and background data across species was compared using paired Mann–Whitney *U* test (**0.001 < *P* < 0.01, ***P < 0.001, NS: not significant). b) Pairwise comparisons of TE content across all windows in the flanking regions of NUMTs/NUMT blocks. The heatmap illustrates the significance (corrected by FDR) of the Mann–Whitney *U* test (yellow: low; white: median; purple: high). The comparisons were performed separately for the windows from the 5 kb upstream and downstream of flanking regions. c) Distribution of genomic loci (intergenic and intronic) in which NUMTs were identified across 36 mammalian genomes. d) Distribution of intergenic and intronic NUMTs/NUMT blocks that are expressed in brain, liver, kidney, and heart samples from platypus. e) Venn diagram showing the number of expressed NUMTs shared amongst brain, liver, kidney, and heart samples from platypus. f) The boxplots indicate the distribution of individual NUMTs that contain intact ORFs, full genic coding regions with stop codons, and incomplete genic coding regions across 45 species.

Previous evidence suggests that NUMTs appear to insert into the genomic locations with rich TE content (mainly retrotransposons; Tsuji et al. 2012). To further examine this association across mammals, we firstly investigated the TE content in 5 kb flanking regions (both upstream and downstream) of each NUMT/NUMT block (see Materials and Methods) with a window size of 500 bp. Although exhibiting different distributions across orders (supplementary fig. S6a, Supplementary Material online), the TE content in the 5 kb flanking regions of NUMTs reveals a distinctive pattern across species using the window-based analysis (Fig. 3a; supplementary fig. S6b, Supplementary Material online). All the windows had a significantly higher TE content than the background average TE content (*P* < 0.05), except the first upstream and downstream windows (Mann–Whitney *U* test; Fig. 3a; see Materials and Methods). These 2 windows, directly connecting with NUMTs, had a significantly lower TE content than the remaining flanking windows up to 5 kb at both ends across species (*P* < 0.05, Mann–Whitney *U* test; Fig. 3b). Aligned with the previous study (Tsuji et al. 2012), we postulated that NUMTs tend not to directly insert into TEs. Based on NUMT sequence identity to the corresponding mtDNA, we also observed that newly inserted NUMTs (higher identity) tend to be located closer to TE than older NUMTs (lower identity). This is evidenced by a negative correlation between NUMT/mtDNA sequence identity and the distance of NUMTs to their closest TE in over two-thirds of mammals (see Materials and Methods; Spearman's correlation test; supplementary fig. S7, Supplementary Material online). All these results indicate that NUMTs are likely found in genomic regions adjacent to TE.

Next, we investigated if NUMTs are prone to be located in introns or intergenic regions. Across the 36 mammals where high-quality genome annotation was available (see Materials and Methods), the number of NUMTs detected in intergenic regions was significantly higher than intronic regions, with the ratio ∼3 to 1 (*P* < 0.001, Mann–Whitney *U* test; Fig. 3c). The pale spear-nosed bat (*Phyllostomus discolor*) has the highest percentage of intergenic NUMTs (89%), whereas the highest percentage of intronic NUMTs (41.9%) is observed in the platypus (*Ornithorhynchus anatinus*; supplementary table S6, Supplementary Material online). While it was estimated that the cumulative sizes of introns and intergenic regions in animal genomes are similar (Francis and Worheide 2017), our result indicates that NUMTs tend to be located in intergenic regions.

### Most NUMTs are not Expressed and Nonfunctional

Upon integration into nuclear genomes, NUMTs are generally considered nonfunctional (Leister 2005; Grau et al. 2020). To confirm this hypothesis, we employed stringent criteria (e.g. junction expression between a NUMT and its flanking regions) to examine NUMT expression in 4 tissue types across 5 species (supplementary fig. S8a, Supplementary Material online; see Materials and Methods). We found that NUMTs are rarely expressed, with a small percentage (0.15% to 4.51%), on average, expressed across different tissues of these species (supplementary fig. S8b, Supplementary Material online). Using the platypus as an example, we observed that expressed NUMTs are significantly enriched in introns (*P* = 0.0136, Chi-square test; Fig. 3d). We speculated that intronic NUMTs can be coexpressed with their host genes without the innovation of independent promoters. This hypothesis may also explain the high percentage of expressed NUMTs seen in platypus (supplementary fig. S8b, Supplementary Material online), as its genome has the highest percentage of intronic NUMTs. It is noteworthy that, using our method, we also identified polymorphic NUMTs that are individual specific in platypus (supplementary fig. S8c, Supplementary Material online), which is commonly seen in mammalian genomes (Dayama et al. 2014, 2020; Wei et al. 2022). In addition, we noticed that 4 NUMTs were expressed across all 4 tissue types in platypus, suggesting that they are under some selective constraints (Fig. 3e). There is a caveat that the percentage of expressed NUMTs across species was underestimated due to NUMT polymorphism across individuals. It is therefore challenging to detect expression of individual-specific NUMTs owing to different sample sources for genome sequencing and RNA-Seq per species. However, we found that on average 99.2% of NUMTs across species contain incomplete open reading frames (ORFs) of mtDNA genes or full genic coding regions with stop codons, based on the nuclear genetic code (Fig. 3f; supplementary table S7, Supplementary Material online). Despite being expressed, they are unlikely to be translated into proteins or, at least, not functioning as protein-coding genes.

### NUMT Presence-and-absence Patterns are Alternative Molecular Markers to Infer Mammal Phylogeny

**Fig. 4.**
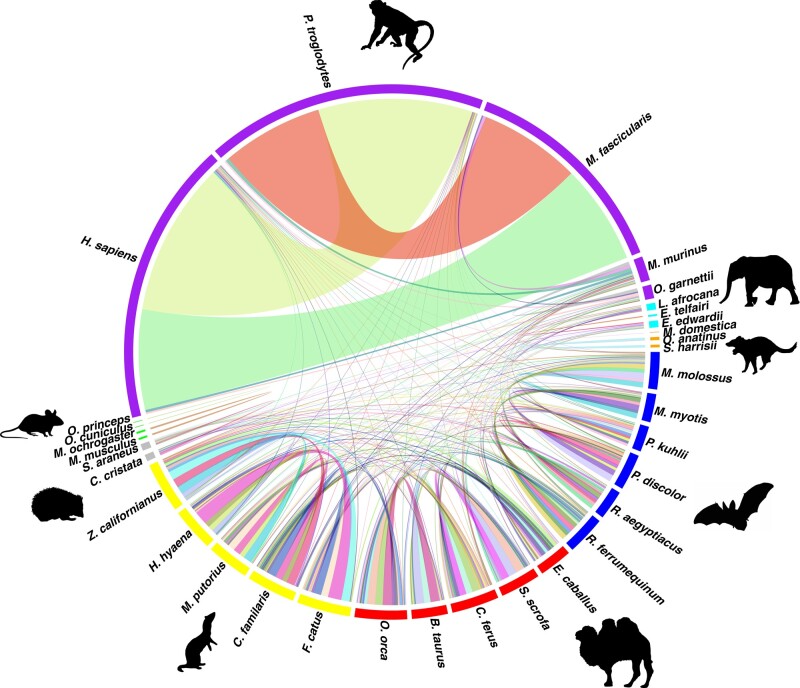
Circos diagram showing the number of predicted orthologous NUMTs/NUMT blocks among 36 species in a pairwise manner. The width of the link between 2 species indicates the number of orthologous NUMTs/NUMT blocks predicted. The color code of the outside layer indicates the species in different defined clades (see Materials and Methods).

**Fig. 5.**
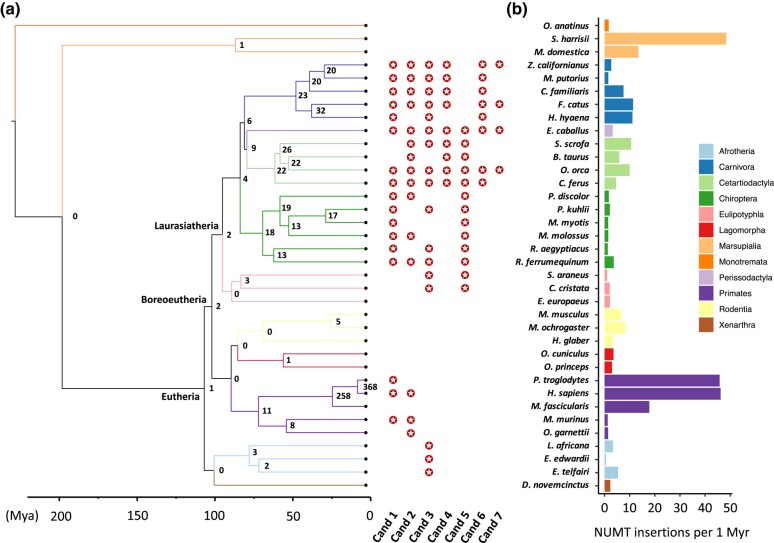
Ancestral NUMTs across mammalian clades and NUMT insertion rate. a) The numbers of ancestral NUMTs/NUMT blocks on the nodes of the phylogenetic tree. The numbers were inferred on the basis of the predicted NUMT orthology relationships across 36 mammals using a coalescent approach. Seven NUMTs/NUMT blocks (Candidates 1 to 7) that were predicted as orthologs in the species across the defined clades are highlighted. b) The estimation of NUMT insertion rates (number of NUMT insertions per 1 Myr) across mammals. For each species, the rate was estimated by dividing the number of individual, nonorthologous NUMTs, the individual NUMTs not found in its closely related species or monophyletic group, by the divergence time (see Materials and Methods).

To investigate the evolutionary trajectories of NUMTs across mammals, we developed a novel method that utilizes protein-coding genes in a conserved genomic synteny block as anchors to locate NUMTs and analyzes their mtDNA origin to infer NUMT orthology between each pair of species (see Materials and Methods; supplementary fig. S9a, Supplementary Material online). The estimation of error probability and expectations (*E*) indicates high accuracy and reliability of our method (see Materials and Methods; supplementary fig. S9b and S9c, Supplementary Material online). By leveraging 630 pairwise comparisons of genome microsynteny among 36 mammals, we observed that only a small proportion of NUMTs/NUMT blocks were predicted to be orthologous in each species, except human (*Homo sapiens*), chimpanzee (*Pan troglodytes*), and macaque (*Macaca fascicularis*; Fig. 4). A relatively large number of orthologous NUMTs/NUMT blocks were found in primates, carnivores, bats, and ungulates, while only a few were identified within the remaining defined clades (see Materials and Methods; supplementary fig. S10, Supplementary Material online). Using phylogenetic patterns of NUMT presence-and-absence, we employed a coalescent approach to predict the NUMTs that are ancestral to each node given the mammal tree (see Materials and Methods). The number of ancestral NUMTs/NUMT blocks predicted on the node decreases as the divergence time increases (Fig. 5a), suggesting that NUMT gain and loss follows an evolutionary pattern and could infer mammal phylogeny. Unsurprisingly, 258 ancestral NUMTs/NUMT blocks were predicted on the node branching to human, chimpanzee, and macaque that diverged only ∼28 Mya (Jebb et al. 2020); 23, 22, 18, and 11 NUMTs/NUMT blocks were, respectively, found ancestral to Carnivora, Cetartiodactyla, Chiroptera, and Primates (Fig. 5a). At the similar divergence time as the above orders in Laurasiatheria, the root of Rodentia was predicted to possess no ancestral NUMTs (Fig. 5a). This is possibly due to the fact that rodent species have undergone a high level of genome reshuffling, whose rates are much greater than other mammalian orders such as Carnivora and Primates (Capilla et al. 2016). These arrangements, such as DNA insertions, inversions, and translocations, disrupt the analogy of genome organization across species so that our microsynteny-based method is not powerful enough to identify orthologous NUMTs that are located in highly reshuffled regions.

Markedly, 7 orthologous NUMTs/NUMT blocks were identified in the species across the defined clades (see Materials and Methods; Fig. 5a; supplementary table S8, Supplementary Material online). Two NUMTs (Candidates 1 and 2) were regarded as ancestral to Boreoeutheria, which were predicted in 17 species across 5 orders and 15 species across 6 orders, respectively (Fig. 5a). We also identified 1 NUMT (Candidate 3) shared by 17 species that lead to the ancestor of Eutheria, but did not find any NUMTs ancestral to the root of mammals (Fig. 5a). These results are not surprising because, as relics of ancient mtDNA, NUMTs evolve under limited selective constraints (Bensasson et al. 2001). Excitingly, we noticed that the phylogenetic patterns of Candidates 4, 6, and 7 support the ancestral clade of Fereuungulata within Laurasiatheria (Fig. 5a), indicating that NUMT presence-and-absence can provide new insights into mammal phylogeny.

### Human, Chimpanzee, and Tasmanian Devil Genomes Exhibit the Highest NUMT Insertion Rates

Assessing NUMT insertion rates in mammals poses a significant challenge, primarily stemming from the complexity of NUMT orthology assignment across species. Using the NUMT orthology relationships and the divergence time within mammals, we estimated the NUMT insertion rate for each species (see Materials and Methods; supplementary table S9, Supplementary Material online). We observed that the species within Marsupialia (Tasmanian devil) and Primates (human and chimpanzee) have higher insertion rates compared to the remaining species (Fig. 5b). An earlier study predicted that NUMT insertion rates in human and chimpanzee were 5.7 and 7.7 NUMTs per 1 Myr, respectively (Hazkani-Covo and Graur 2007), whereas we estimated much higher insertion rates (46.2 and 45.8 NUMTs per 1 Myr for human and chimpanzee, respectively; Fig. 5b). The disparities lie mainly in the fact that we predicted nearly twice as many NUMTs (846 in human and 819 in chimpanzee; Fig. 1a) as the numbers in that study [452 and 469 in human and chimpanzee respectively (Hazkani-Covo and Graur 2007)], and a higher percentage of species-specific NUMTs [27.4% as opposed to 15% (Hazkani-Covo and Graur 2007)] whose insertions are deemed to occur after their speciation.

### Caution Should be Taken When Using Ancestral NUMT Trees to Infer Phylogeny

**Fig. 6.**
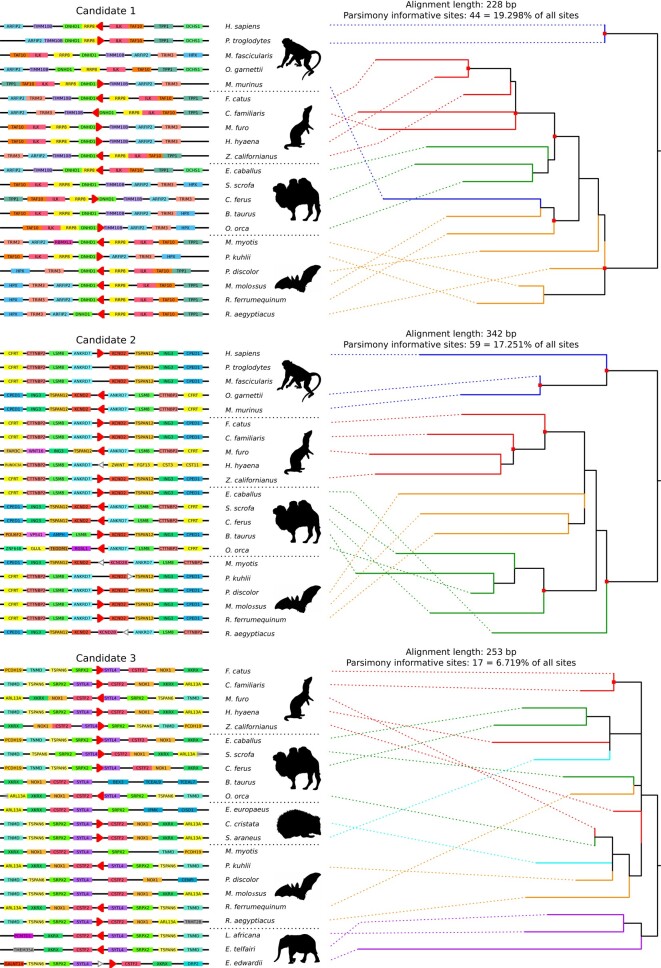
Visualization of the genome microsynteny of 3 ancestral NUMTs (Candidates 1 to 3) predicted across the defined clades and their phylogenetic trees. For each candidate, the lines represent the conserved microsynteny blocks across species. Protein-coding genes are shown as rectangles on the lines. Genes with no gaps between each other indicate that they have overlapping genomic coordinates. NUMTs are represented by triangles, with its direction relative to the corresponding mtDNA indicated. Red triangles represent ancestral orthologous NUMTs, while white ones represent NUMTs that are considered as nonorthologous. The triangles that overlap with protein-coding genes indicate that the NUMTs are located in the intronic regions of these protein-coding genes; otherwise, the NUMTs are located in intergenic regions or 3′-UTRs. The phylogenetic tree for each NUMT candidate was constructed using an ML approach. The effective alignment length and the number of parsimony informative sites for each tree are indicated on the plot. The dots on the nodes of the trees indicate high branch supports (HS-aLRT > 90%, aBayes > 0.9, and UFBoot > 90%). The dashed lines connect the species and the branches they belong to.

To ascertain if alignments of ancestral NUMTs are appropriate to infer phylogenetic relationships, we used a maximum-likelihood (ML) method to construct phylogenetic trees for 3 orthologous NUMTs (Candidates 1 to 3) predicted across distant orders (see Materials and Methods). We observed that the trees inferred from Candidates 1 and 2 are highly similar to the mammal tree (Jebb et al. 2020), with most species in the same order grouped together (Fig. 6). The exceptions lie in *Microcebus murinus* and a few bat species in Candidate 1 and *Equus caballus* in Candidate 2. For the tree inferred from Candidate 3, the 2 main clades Boreoeutheria and Atlantogenata are unambiguously split but the phylogenetic relatedness within Boreoeutheria is largely unresolved (Fig. 6). Therefore, phylogenies inferred from ancestral NUMTs should be interpreted with caution.

## Discussion

In this study, we systematically investigated the characteristics of NUMT insertions in 45 mammalian genomes, and determined their radiation, genomic distribution, mtDNA origin, functionality, and insertion rates across species. The first requirement is to ensure the reliability of our NUMT predictions. While it is impractical to experimentally validate all predicted NUMTs due to the large number across species and their polymorphism across individuals, a few alternative validation methods exist. The recent study on human NUMTs employed the whole genome alignments that included thousands of human individual genomes to validate their NUMT predictions (Wei et al. 2022). However, this approach does not apply to our study that involves 45 mammalian species representing over 200 million years of evolution. Instead, we found an array of evidence demonstrating the authenticity of our NUMT predictions. The evidence includes a degree of sequence divergence between NUMTs and their mtDNA counterparts, the support by the PacBio raw reads for genome assembly, the presence of stop codons in most NUMTs, and gain-and-loss phylogenetic patterns of orthologous NUMTs. Although one should be cautious when interpreting recently inserted NUMTs, our analyses indicate that our predicted NUMTs are authentic and reliable.

By analyzing NUMT coverage in mtDNA with a 50 bp sliding window, we revealed that the mtDNA regions from which NUMTs originate are not random (Fig. 2a–c). It will be interesting to see if the current conclusion stands when more species are included in the analyses. Additionally, at single-nucleotide resolution, the coverage of NUMTs in mtDNA exhibits a species-specific pattern. Consistent with the previous study (Tsuji et al. 2012), we noticed that some regions within the D-loop, mainly the heavy strand region, seldom produce NUMTs in most of the species in Primates (supplementary fig. S5b, Supplementary Material online). Nevertheless, a large percentage of NUMTs derived from mtDNA D-loop was observed in several species, such as cat, rat, American pika, and Tasmanian devil, representing different phylogenetic orders (supplementary fig. S5b, Supplementary Material online). Our results suggest that the NUMT origination from mitogenomes is not completely stochastic and also support the current hypothesis that NUMT insertion results from the degradation of abnormal mitochondria in which mitogenomes are randomly sheared (Kleine et al. 2009). It is noteworthy that we analyzed the NUMT coverage in mtDNA up to 16,000 bp, which only covers part of the D-loop region. Due to the complex features of the D-loop (e.g. length differences across species; species-specific tandem repeats), the NUMT coverage in the D-loop region needs to be further explored.

We also demonstrated that NUMTs are likely found in transposon-rich and intergenic regions (Fig. 3a–c). Unlike transposable elements, NUMTs do not have the mechanisms to duplicate and translocate themselves independently, and ample studies (Tsuji et al. 2012; Michalovova et al. 2013; Wang et al. 2020), including our analyses (supplementary fig. S4, Supplementary Material online), reported a positive correlation between NUMT content and genome TE content. In addition, it is important to note that the genomic regions into which NUMTs insert might be random, but these regions may be immediately subject to certain selective constraints, such as purifying selection, so that newly inserted NUMTs may be quickly eliminated. Therefore, our result is not surprising as introns are broadly more functionally conserved than intergenic regions due to the enrichment of *cis*-regulatory elements in introns, such as intronic splicing enhancers and silencers (Chorev and Carmel 2012; Shaul 2017). Disruptions of these regulators and exon–intron splice junctions via NUMT insertion may alter gene expression or produce dysfunctional proteins, leading to detrimental consequences (Turner et al. 2003; Goldin et al. 2004).

It is methodologically challenging to assign NUMT orthology across mammals owing to the fast evolution of NUMTs and their flanking regions, so that traditional alignment-based approaches are not feasible (Hazkani-Covo 2009; Hazkani-Covo et al. 2010). To address this challenge, we established a novel, synteny-based and accurate method to assign NUMT orthology relationships between species. By constructing the ancestral state of NUMTs, we revealed that the presence-and-absence patterns of 3 NUMT blocks (Candidates 4, 6, and 7) support the ancestral clade of Fereuungulata (Fig. 5a). Currently, the interordinal relationships of the laurasiatherian mammals still remain controversial, as multiple phylogenomic studies gave rise to alternative topologies (Zhou et al. 2012; Foley et al. 2016). The major challenge is ascribed to the difficulty resolving short internal branches that connect the 4 key clades [Cetartiodactyla, Perissodactyla, (Carnivora + Pholidota), Chiroptera] that radiated in the late Cretaceous period (Springer et al. 2003). Our result disagrees with the earlier mammalian phylogenies (using a supermatrix of 26 gene fragments across 164 mammals) which placed bats as the sister clade to ungulates (((Perissodactyla + Cetartiodactyla), Chiroptera), Carnivora) (Meredith et al. 2011), but supports the recent study (using a supermatrix of 12,931 genes across 48 mammals) that inferred a sister-group relationship between carnivores and ungulates (((Perissodactyla + Cetartiodactyla), Carnivora), Chiroptera) (Jebb et al. 2020). As a perspective of NUMT evolution, our results imply that NUMT presence-and-absence patterns could be an alternative means to infer mammal phylogeny and provide new insights into the resolution of controversial nodes. The power of this approach is expected to increase when more high-quality genomes and annotations become available, so that more fixed, orthologous NUMTs are likely to be recovered amongst closely related species.

Using the inferred ancestral NUMTs on the nodes of the mammal tree and the divergence time, we estimated the NUMT insertion rate for each species (Fig. 5b). However, our insertion rates were overestimated because we were unable to identify NUMTs that originated from post insertion duplication events. This is due to the intrinsic complexity of NUMT duplication (e.g. tandem duplication; segmental intrachromosomal and interchromosomal duplication; Woischnik and Moraes 2002) and NUMT features (e.g. short length; homology to each other; selectively unconstrained; Leister 2005). Lack of chromosome-level genome assemblies has also hindered the identification of these events. We attempted to find tandem duplicated NUMTs that are located within 10 kb windows in genomes, but only a small number of NUMTs meet this criterion (see Materials and Methods; supplementary table S2, Supplementary Material online). As such, we decided not to consider them when calculating insertion rates. To obtain more precise rates, it is therefore of great importance to analyze multiple chromosome-level genomes across a wide range of species to determine NUMT duplication levels. It was estimated that up to 85% of NUMTs in the human genome originated from insertion events (Hazkani-Covo and Graur 2007). Even though evidence for large segmental duplications exists in many species (Bensasson et al. 2003; Triant and DeWoody 2007), this seems to be rare (Hazkani-Covo and Covo 2008). Together with our analyses on tandemly duplicated NUMTs, we hypothesize that NUMT bursts in the human, chimpanzee, and Tasmanian devil genomes may result from rapid insertion rather than post insertion duplication. However, the underlying reasons for these expansions are currently not well understood. It is speculated that the increase of NUMT insertion can be attributed to changes in environmental factors (Wang et al. 2012). It has been proven that yeast cultured under nonoptimal temperatures demonstrated an accelerated rate of mtDNA escape to the nucleus genome (Cheng and Ivessa 2012). For marsupials, it was hypothesized that the expansion of NUMTs in Dasyuridae may result from the rapid drop of global temperature, an event known as the Miocene climate transition that occurred shortly after the divergence of Dasyuridae (Kealy and Beck 2017). However, this conjecture is weakened by the paucity of NUMTs found in species with a similar divergence time.

Although NUMT gain-and-loss patterns over evolutionary time can provide new insights into mammal phylogeny, one should be cautious when using ancestral NUMT trees to infer phylogenetic relationships. These ancestral NUMTs may be subject to different levels of selective constraints in respective species over long evolutionary time. The erroneous groupings in the trees inferred from Candidates 1 and 2 (Fig. 6) are not unexpected given the short fragments analyzed and the fact that several sequence pairs are only overlapping by less than 100 bp. Candidate 3, the NUMT ancestral to Eutheria, was derived from a short fragment of the mtDNA D-loop region which spans a conserved block, and the NUMT sequences have also remained conserved despite ∼100 million years of evolution (only 17 parsimony informative sites across 17 species, Fig. 6). One could combine the information from multiple NUMT alignments to infer relationships, but it is challenging to obtain a consequent number of NUMTs for inferring interordinal relationships in mammals (Fig. 5). Given these limitations, interordinal relationships inferred from ancestral NUMT sequences should be interpreted with caution. That being said, although beyond the scope of our work, more recent NUMTs could be interesting phylogenetic markers to infer intraordinal relationships.

To our knowledge, this is one of the most comprehensive studies that explored the characteristics of NUMTs and their evolutionary trajectories in a mammal-wide context. Over the past 200 million years, NUMTs have undergone a fast birth and death in mammalian genomes. Although the reason for their expansions in a few species remains unclear, we revealed that NUMTs are derived from nonrandom regions in mtDNA, are likely found in TE-rich and intergenic regions, and unlikely code for functional proteins. Using the new synteny-based method we established, we further demonstrated that NUMT presence-and-absence patterns can provide new insights into mammal evolution, while phylogenies inferred from ancestral NUMTs should be interpreted with caution. As opposed to the traditional alignment-based methods, our novel approach enables NUMT orthology assignment among distantly related species, providing an alternative means to study phylogeny. This method can potentially be utilized to predict orthology relationships of NUMTs across different taxa or other fast-evolving, noncoding DNA, such as transposable elements. In the future, a comprehensive taxonomic sampling of species with multiple high-quality individual genomes and a refined genome microsynteny atlas across species will be required to gain a full blueprint of NUMT evolution in mammals. Uniquely, our study broadens the current knowledge on the characteristics of NUMT integrations in mammalian genomes and highlights the merit of NUMT evolution in phylogenetic inference.

## Materials and Methods

### Genome Sampling

Forty-five published mammalian genomes were used to investigate the evolution of NUMTs in mammals. The list of species comprises Monotremata (*n* = 1), Marsupialia (*n* = 4), Afrotheria (*n* = 4), Xenarthra (*n* = 1), Lagomorpha (*n* = 2), Rodentia (*n* = 5), Scandentia (*n* = 1), Primates (*n* = 6), Eulipotyphla (*n* = 3), Pholidota (*n* = 1), Carnivora (*n* = 5), Chiroptera (*n* = 6), Perissodactyla (*n* = 2), and Cetartiodactyla (*n* = 4). These species represent the vast ecological and evolutionary diversity within mammals, representing over 200 million years of evolution. We included chicken *Gallus gallus* as a nonmammal outgroup. We prioritized the latest assembly version available for each species when selecting genomes for our analyses (April 2020). The quality of genomes was assessed by BUSCO (v4.0; Waterhouse et al. 2018) and the average genome completeness is 94.0% ± 3.0%, indicative of high quality of these assemblies. The detailed information, including species, genome versions, and genome statistics, is available in supplementary table S1, Supplementary Material online.

### Optimization of NUMT Identification Pipelines

For each species, we employed a local BLAST approach (Altschul et al. 1990) to identify NUMT insertions by querying its nuclear genome using the corresponding complete mitochondrial genome sequences. Where the mitogenome of the same species was not available, that of its closely related species was used (supplementary table S1, Supplementary Material online). To facilitate BLAST search, circular mitogenomes were presented as linear sequences that begin with tRNA-Phe and end with D-loop.

There are currently no standard pipelines and criteria to define NUMT insertions. To best profile NUMTs in mammalian genomes, we tested BLASTN (v2.9.0), mega-BLAST, and discontinuous mega-BLAST (more sensitive to detect divergent sequences) with a combination of 3 key parameters (*-template_length*; *-template_type*; *-word_size*) using the human genome (hg38) as an example (supplementary table S10, Supplementary Material online). We applied an *E*-value threshold (10^−3^) and a minimum HSP (high-scoring segment pair) length (30 bp) to avoid potential false positives that were derived from assembly errors or nonmitochondrial origin. Mega-BLAST only produced 186 HSPs, with many old NUMTs (less than 80% identity to their corresponding mtDNA) undetected. In contrast, BLASTN and discontinuous mega-BLAST consistently reported more NUMTs, and different combinations of parameters yielded similar HSP numbers (839 ± 8) which are higher than most of previous studies reported (Hazkani-Covo and Graur 2007; Hazkani-Covo 2009; supplementary table S10, Supplementary Material online). Based on the sensitivity, we chose discontinuous mega-BLAST with the following parameters: *-template_length 18*, *-template_type optimal*, and *-word_size 11* as the optimal approach.

### Identification of NUMTs in 46 Genomes

Using the optimal method, we predicted NUMTs in 45 mammalian genomes and the *G. gallus* genome. For each species, the raw BLAST HSPs were merged if they meet the following conditions: (i) there is a less than 10 bp gap in the nuclear genome between 2 adjacent HSPs that have continuous mitogenome coordinates correspondingly; (ii) a single NUMT that traverses the D-loop region are split into 2 HSPs due to the boundary created by linearization of circular mtDNA. We further removed the HSPs located in very short contigs (<20 kb) as they were likely the result of mtDNA contamination or assembly errors. Subsequently, for each species, we assigned continuous numbers (e.g. Hsap_numt_1) to the processed HSPs according to their nuclear genomic coordinates (supplementary table S3, Supplementary Material online).

### Authenticity of NUMT Prediction

To confirm the authenticity of our predictions, we firstly investigated NUMT sequence divergence from their mtDNA counterparts by assessing the distribution of NUMT identity to the corresponding mtDNA across species. To account for heteroplasmy amongst mtDNA, we used a 98% sequence similarity as the threshold to demonstrate NUMT sequence divergence from their mtDNA counterparts. NUMTs (<98% identity) are unlikely to be artifacts introduced during genome assembly, while it is challenging to distinguish recent NUMTs (>98% identity) from true mtDNA without Illumina or PacBio raw reads for genome assembly.

To further show the divergence between NUMTs and mtDNA, we built 2 phylogenetic trees using NUMT sequences across 45 species. We obtained all NUMT sequences that were mapped to 2 mtDNA loci, Cytochrome b (*CYTB*), and NADH dehydrogenase 1 (*ND1*), the 2 genes which have high NUMT coverage and are commonly used in phylogenetic studies (Zardoya and Meyer 1996; Morgan et al. 2014). For each locus, we only selected the mapped NUMT sequences that are equal or over 500 bp (188 sequences for *CYTB* and 215 sequences for *ND1*), and aligned them together with mtDNA of 45 species using MAFFT (Katoh and Standley 2013). The appropriate nucleotide substitution models (GTR + F + G4 for *CYTB* and K3Pu + F + R6 for *ND1*) were selected based on the Bayesian information criterion using ModelFinder (Kalyaanamoorthy et al. 2017; supplementary table S11, Supplementary Material online). Next, we inferred the ML tree using the partition model in IQ-TREE (Nguyen et al. 2015; Chernomor et al. 2016) for each locus. To search for the best-scoring ML, we performed ultrafast bootstrap (UFBoot; Hoang et al. 2018) with 1,000 bootstraps and 1,000 topology replicates. To verify the robustness of the ML trees, the branch supports were evaluated using SH-like approximate likelihood ratio test (SH-aLRT; Guindon et al. 2010) and a Bayesian-like transformation of aLRT (aBayes; Anisimova et al. 2011). SH-aLRT was performed with 1,000 replicates. The ML, SH-aLRT, and aBayes analyses were performed using W-IQ-TREE (Trifinopoulos et al. 2016). The trees were rooted by Monotremata.

In addition, we also employed the PacBio raw reads we published for the genome assembly of the 6 bat species (Jebb et al. 2020) used in this study to assess the reliability of our NUMT predictions. We were unable to implement this method to validate the NUMTs predicted from the remaining species owing to unavailability of raw reads for their genome assembly. We aligned the NUMTs predicted in each bat species against the PacBio raw reads using the optimal BLAST approach aforementioned. NUMTs were considered “real” if they were located within PacBio raw reads where the flanking regions of the NUMT loci were mapped to the nuclear genome. The coverage of PacBio reads at the junctions between NUMT and nuclear DNA was determined by averaging the number of PacBio reads spanning the 5′ and 3′ ends for each NUMT.

### Characteristics of NUMT Prediction

We firstly investigated the distribution of NUMT length across species. NUMT length distributions were compared across species in a pairwise manner using Kolmogorov–Smirnov test. *P*-values obtained from the tests of within-order and across-order comparisons were log_10_ transformed and were further compared using a Mann–Whitney *U* test. Next, we performed correlation analyses between some NUMT characteristics (number; accumulative length) and genome statistics (genome size; scaffold number; TE content) using Spearman's correlation test. Significances of the tests were further corrected by phylogeny using the *phytools* R package (Revell 2012). The time-calibrated phylogenetic tree required for the phylogeny correction was obtained from the recently published mammal phylogeny (Jebb et al. 2020). Next, we examined if there are any “hotspots” or overabundance in mtDNA from which NUMTs were derived. To do this, we obtained the mitochondrial cross coverage of NUMTs across species using *genomecov* in the BEDTools suite (v2.30.0; Quinlan and Hall 2010). We firstly scanned the coverage of mtDNA with a 50 bp sliding window and calculated the median coverage per window for each species. Then, we conducted comparisons of the coverage between all possible windows across species using Mann–Whitney *U* test. Due to the disparity in mtDNA length across species, we only analyzed the first 320 windows representing 16,000 bp in mtDNA (1st to 16,000th bp), starting with the gene tRNA-Phe. Furthermore, we identified the over-represented mtDNA genic regions by NUMTs for each species. In brief, we transformed the coverage data, representing coverage per base on mtDNA, into *z*-scores. The bases with coverage values deviated by more than 3 SD (*z*-score > 3) from the norm within the coverage distribution were identified as regions of over-representation by NUMTs (supplementary table S5, Supplementary Material online).

To further confirm our results, we simulated a null distribution of mtDNA coverage by randomly reshuffling mitogenome coordinates of NUMTs for each species, performed pairwise comparisons of the coverage between all windows as mentioned above, and repeated these analyses 1,000 times. For the coordinates, we only randomly picked a start position for each NUMT (between 1 and 16,000) and used the length of observed NUMTs to calculate the end coordinates. Hence, the length distribution of NUMTs was identical between the observed and simulated datasets. In order to take mtDNA circularity into account, coordinates greater than 16,000 (e.g. 16,200 to 16,450) were subtracted 16,000 (e.g. leading to 200 to 450). Finally, the number of significant tests obtained from the observed dataset was compared to the distribution obtained from the 1,000 simulated datasets (i.e. null distribution if coverage was homogenous).

### Assembly of NUMT Blocks

To facilitate the downstream analyses, for each species, the adjacent NUMTs with a nuclear genomic distance less than 2 kb were assembled as a single NUMT block, regardless of their orientations in the mitogenome (supplementary table S4, Supplementary Material online). NUMT blocks are considered complex if they are composed of 3 or more individual NUMTs. To demonstrate the reliability of predicted large NUMT blocks, as an example, we examined their nuclear genomic loci using our published PacBio raw reads for *Molossus molossus* bat genome assembly (supplementary fig. S3d, Supplementary Material online), given that *M. molossus* possesses one of the largest NUMT blocks amongst the 6 bat species we studied.

### Analyses of NUMT Insertion “hotspots” in Nuclear Genomes

To understand if there are any “hotspots” or preferences in nuclear genomic regions in which NUMT insertions occurred, we firstly investigated the TE content in the flanking regions of NUMTs/NUMT blocks in genomes. For each species, we extracted 5 kb flanking sequences, both upstream and downstream, of each NUMT/NUMT block using *getfasta* in the BEDTools suite (v2.30.0; Quinlan and Hall 2010). Their TE contents were estimated using RepeatMasker (v4.1.2; Smit 2013–2015) with a window size of 500 bp. To establish a comparative baseline for TE content at the species level, we employed the following method. For each NUMT, we randomly selected new genomic coordinate while maintaining the original NUMT length. During this process, we excluded the terminal regions (5 kb) of scaffolds, ensuring that each “pseudo-NUMT” retained a minimum of 5 kb of flanking regions on both sides. Subsequently, we calculated the average percentage of TEs within the 5 kb upstream and downstream flanking regions of all “pseudo-NUMTs” using a 500 bp window size. The procedure above was iterated 1,000 times, and the average TE content within each of 20 windows was computed. For each window, we compared the TE content between the observed and background data across species using paired Mann–Whitney *U* test (supplementary table S12, Supplementary Material online).

Using this statistical method, we also compared the TE content between different windows in a pairwise manner. It is noted that TE content may be underestimated because the TE database is currently biased for only a few model species such as human and mouse (Smit 2013–2015). Because TE were compared across windows of NUMT flanking regions within species, this bias is unlikely to affect our conclusions. To further explore if newly inserted NUMTs are located in proximity to TE, for each species, we performed correlation analyses between NUMT/mtDNA sequence identity and the distance of NUMTs to their closest TE (averaged by both ends) using Spearman's correlation test. Owing to the heterogeneity in sequence identity of individual NUMTs, NUMT blocks were excluded from this analysis.

Next, we investigated if NUMTs are likely located in intronic or intergenic regions. To achieve this, we obtained high-quality genome annotation files published along with the genomes from the National Center for Biotechnology Information. We did not include the following 9 species: *Rattus norvegicus*, *Sciurus vulgaris*, *Saimiri boliviensis*, *Manis javanica*, *G. leadbeateri*, *Ceratotherium simum*, *T. manatus*, *Tupaia belangeri*, and *V. ursinus* in the analysis. This is because the annotation files for *G. leadbeateri*, *S. vulgaris*, and *M. javanica* were not publicly available, while the protein-coding gene annotation for the 6 remaining species was only confined to gene models and loci without the assignment of associated “gene symbols”. For the remaining 36 species, noncoding gene annotations were removed from their genome annotation files. Intronic and intergenic NUMTs were determined by merging their genomic coordinates with protein-coding gene coordinates using *merge* in the BEDTools suite (v2.30.0; Quinlan and Hall 2010). However, there is a caveat that intergenic NUMTs close to protein-coding genes might be located in gene untranslated regions (UTRs). This is because UTRs are typically not well annotated in most mammalian genomes.

### Functional Predictions of NUMTs

To ascertain if NUMTs are expressed and functional, we obtained and analyzed publicly available RNA-Seq data of 4 tissue types (brain, kidney, liver, and heart) from 5 species (human, naked mole-rat, cow, dog, and platypus; supplementary table S13, Supplementary Material online). Because NUMT sequences could be very similar to their corresponding mtDNA sequences depending on their insertion time, we used the stringent criteria to determine if a NUMT is expressed. NUMTs are considered expressed if (i) at least 2 RNA-Seq reads support the junctions between NUMTs and their flanking nuclear genomic regions with at least 5 bp overhangs and (ii) the coverage of NUMTs by RNA-Seq reads is >70%. To achieve this, for each species, we extracted the sequence of each NUMT/NUMT block together with their 200 bp upstream and downstream flanking sequences as references using the same method mentioned above. Prior to NUMT quantification, adaptors and low-quality regions (base score < Q25) in raw RNA-Seq reads were filtered using cutadapt (v3.5; Martin 2011). We then mapped the clean reads from each sample to the corresponding references using HISAT2 (v2.2.1; Kim et al. 2015). NUMT expression was analyzed using Samtools (v1.13; Li et al. 2009) and the BEDTools suite (v2.30.0; Quinlan and Hall 2010), and was further visualized in the genome browser IGV (v2.14.1; Robinson et al. 2011). Next, we explored if NUMT sequences have the potential to be translated into proteins. To achieve this, we investigated all 17,732 individual NUMTs across 45 mammals. We firstly identified NUMTs that contain the entire regions of any mtDNA protein-coding genes and analyzed their ORFs using Geneious (v11.0.5; https://www.geneious.com). ORFs were then translated into proteins based on both nuclear and mitochondrial genetic codes (supplementary table S7, Supplementary Material online).

### A Novel Method to Determine NUMT Orthology Between Distant-related Species

We initially attempted to align NUMT sequences along with 1 kb flanking sequences at each end in each species using MAFFT (Katoh and Standley 2013). With the exception of the closely related human and chimpanzee which diverged only ∼5 Myr ago, NUMTs with flanking regions were poorly aligned amongst the remaining species (data not shown), and thus, the results were not conclusive. As such, traditional alignment-based methods are not feasible to infer orthologous NUMTs between distant-related species.

To address this problem, we established an innovative and practical approach that utilizes genome microsynteny to identify orthologous NUMTs within mammalian clades. We used protein-coding genes in a conserved genomic synteny block as anchors to infer orthologous NUMTs/NUMT blocks among species. We regarded 2 NUMTs/NUMT blocks from respective species as orthologous, if (i) they are located in the same synteny block within a distance of 6 protein-coding genes (3 genes upstream and 3 genes downstream of a NUMT/NUMT block) and (ii) their sequences overlap with each other by at least 50% (supplementary fig. S9a, Supplementary Material online). With these criteria, we evaluated the probability (error rate) that 2 NUMTs/NUMT blocks from respective species were assigned as orthologs by chance. The size of a synteny block (6 protein-coding genes) was selected to allow the detection of orthologous NUMTs in slightly rearranged genomic regions. Due to the complexity of NUMT insertions (e.g. different NUMT lengths; complicated NUMTs) and different characteristics of genomes (e.g. different numbers of protein-coding genes), we employed a simplified formula to estimate the error rate of NUMT orthology assignment for each pair of species (supplementary fig. S9b, Supplementary Material online). Suppose that the average number of protein-coding genes in mammalian genomes is 20,000, that the average length of NUMTs is 200 bp, and that the average size of mammalian mitochondrial genomes is 16,600 bp. The error rate was calculated at 3.03 × 10^−6^ (see supplementary fig. S9b, Supplementary Material online for the explanation). The mathematical error expectation (*E*) of orthology assignment was estimated by multiplying the error rate (3.03 × 10^−6^) by the total number of all possible NUMT pairs (*N_a_* × *N_b_*) between 2 species (supplementary fig. S9c, Supplementary Material online). *N_a_* and *N_b_* stand for the number of NUMTs/NUMT blocks in species A and B, respectively. The error expectation (*E*) indicates the number of false-positive NUMTs regarded as orthologs (by random chance) between 2 species given the number of their all possible NUMT pairs and our criteria for NUMT orthology assignment. With the exception of the comparisons between *S. harrisii* and the other species, the estimated error expectations (*E*) amongst the remaining species are far below 1 (supplementary fig. S9c, Supplementary Material online). These results imply that our novel approach is accurate and feasible to assign NUMT orthology between distantly related species within mammals.

### Determination of Orthologous NUMTs Across Mammals

Using this method, we predicted orthologous NUMTs/NUMT blocks between species by leveraging 630 pairwise comparisons of genome-wide microsynteny across 36 mammals in which high-quality gene annotation files are available. For each species, we integrated the predicted NUMT annotations with the protein-coding gene annotations using *merge* in the BEDTools suite (v2.30.0; Quinlan and Hall 2010), and assigned orthologous NUMTs/NUMT blocks between 2 species on the basis of the above criteria. Each predicted pair was manually inspected. The NUMT orthology between 2 species was visualized using the R package *circlize* (v0.4.15; Gu et al. 2014), and the NUMT orthology networks were established using the R package *UpSetR* (v1.4.0; Conway et al. 2017). To facilitate data visualization and interpretation, we categorized these 36 species into 8 clades with a balanced species number per clade. These defined clades include Clade 1 (Monotremata + Marsupialia, *n* = 3), Clade 2 (Afrotheria + Xenarthra, *n* = 4), Clade 3 (Primates, *n* = 5), Clade 4 (Rodentia + Lagomorpha, *n* = 5), Clade 5 (Eulipotyphla, *n* = 3), Clade 6 (Carnivora, *n* = 5), Clade 7 (Perissodactyla + Cetartiodactyla, *n* = 5), and Clade 8 (Chiroptera, *n* = 6). It is noteworthy that we predicted 6 orthologous NUMTs/NUMT blocks between *O. anatinus* and *S. harrisii* (Clade 1; supplementary fig. S10a, Supplementary Material online). Given their long divergence time and our estimation of the error expectation (*E* = 2.99; supplementary fig. S9c, Supplementary Material online), it is likely that these predictions are false positives mainly because *S. harrisii* has the largest number of NUMTs (Fig. 1a).

### Ancestral Orthologous NUMTs on the Phylogenetic Tree and NUMT Insertion Rates

Because most NUMTs are nonfunctional and under limited selective constraints, we employed a simple coalescent method to infer ancestral NUMTs/NUMT blocks on the nodes of the given phylogenetic tree (Jebb et al. 2020) using the phylogenetic patterns of NUMT presence-and-absence. A NUMT/NUMT block is regarded as ancestral on the node if it is identified as orthologous across species in both bifurcating clades to which the node branches. Next, we estimated the NUMT insertion rate as described in Hazkani-Covo (2009). For each species, we obtained the number of individual NUMTs that do not have orthologs in its most closely related species or monophyletic group. The NUMT insertion rate (number of insertions per 1 million years) for each species was estimated by dividing the number of individual, nonorthologous NUMTs by the divergence time. It is noteworthy that it is particularly challenging to identify duplications of preexisting NUMTs due to the complexity of these events and lack of chromosome-level genome assemblies. We attempted to identify tandem duplicated NUMTs which are located within 10 kb windows, have similar start or end mitogenome coordinates (±10 bp), and overlap with each other by at least 50% for each species. These duplicated NUMTs were further verified by aligning them along with 1 kb flanking sequences at both ends using MAFFT (Katoh and Standley 2013). Due to the scarcity of this case observed across mammals (supplementary table S1, Supplementary Material online), we decided not to consider duplication events when calculating the insertion rate.

### Alignments of Ancient Ancestral Orthologous NUMTs Across Species

Next, we visualized the genomic positions of all the 7 ancestral NUMTs/NUMT blocks in genomic microsynteny across species (Fig. 6; supplementary fig. S11, Supplementary Material online) and constructed 3 phylogenetic trees for the individual ancestral NUMTs (Candidates 1 to 3), respectively. The sequences of Candidates 1 to 3 were aligned and trimmed to the shortest common length using Gblocks (v0.91b; Castresana 2000). The phylogenetic trees were inferred and verified as extensively described above. For Candidates 1 to 3, the alignment lengths are 228 bp (17 sequences), 342 bp (15 sequences), and 253 bp (17 sequences), respectively. The best-fit models are HKY + F + I, TPM3 + F, and TPM2u + F, respectively.

### Statistical Analyses

The statistical analyses used in this study, including Mann–Whitney *U* test, Kolmogorov–Smirnov test, Spearman's correlation test, and Chi-square test were performed in R (v4.1.1; Team 2014). *P*-values were corrected by multiple tests using FDR where applicable. Statistical tests with corrected *P* < 0.05 were considered significant unless specifically defined.

## Supplementary Material

msad278_Supplementary_Data

## Data Availability

The publicly available genome, mitogenome, and RNA-Seq data used in this study are documented in the supplementary tables S1 and S13, Supplementary Material online. The intermediate data supporting the conclusions can be available at the GitHub page (https://github.com/huangzixia/NUMT_evolution_in_mammals).
